# Rheumatism and chronic fatigue, the two facets of post-chikungunya disease: the TELECHIK cohort study on Reunion island

**DOI:** 10.1017/S0950268818000031

**Published:** 2018-02-28

**Authors:** A. Duvignaud, A. Fianu, A. Bertolotti, J. Jaubert, A. Michault, P. Poubeau, A. Fred, M. Méchain, B.-A. Gaüzère, F. Favier, D. Malvy, P. Gérardin

**Affiliations:** 1 Department of Tropical Medicine and Clinical International Health, Division of Infectious Diseases and Tropical Medicine, CHU Bordeaux, Bordeaux, France; 2 Infectious Diseases in Low Income Countries (IDLIC), Bordeaux Population Health Research Centre (INSERM U1219, Université de Bordeaux, ISPED), Bordeaux, France; 3 INSERM CIC 1410, CHU Réunion, Saint Pierre, Reunion; 4 Department of Infectious Diseases, CHU Réunion, Saint Pierre, Reunion; 5 Bacteriology, Virology and Parasitology lab, CHU Réunion, Saint Pierre, Reunion; 6 Department of Social and Preventive Medicine, School of Public Health, Montreal University, Montreal, Canada; 7 Polyvalent Intensive Care Unit, CHU Réunion, Saint Denis, Reunion; 8 UM 134 PIMIT Processus infectieux en Milieu Insulaire Tropical (Université de La Réunion, CNRS 919, INSERM U 1187, IRD 249), CYROI, Sainte Clotilde, Reunion

**Keywords:** Alphavirus, chikungunya, chronic fatigue, cohort study, rheumatic disease

## Abstract

Prolonged fatigue is increasingly reported among chikungunya virus (CHIKV)-infected populations. We investigated the relationships between CHIKV exposure, long-lasting rheumatic musculoskeletal pain (LRMSP) and chronic fatigue. 1094 participants (512 CHIKV seropositive and 582 seronegative) of the TELECHIK population-based cohort were analysed considering the duration of the manifestations throughout an average 2-year follow-up. Weighted prevalence rates and prevalence ratios for LRMSP, idiopathic chronic fatigue (ICF), and chronic fatigue syndrome (CFS)-like illness, both latter syndromes adapted from Centers for Disease Control (CDC)-1994/Fukuda criteria, were compared. Population attributable fractions (PAF) were estimated to assess the contribution of CHIKV infection to each of the three phenotypes. Among 362 adult subjects who had reported either rheumatic pain or fatigue at the onset of the infection, weighted prevalence rates of LRMSP, ICF and CFS-like illness were respectively of 32.9%, 38.7% and 23.9%, and of 8.7%, 8.5% and 7.4% among initially asymptomatic peers (*P* < 0.01, respectively). Each of the three outcomes was highly attributable to chikungunya (PAF of 43.2%, 36.2% and 41.0%, respectively). In the sub-cohort of CHIKV-infected subjects, LRMSP, ICF and CFS-like illness, which overlapped in 70%, accounted for 53% of the chronic manifestations. In addition to rheumatic disease, chronic fatigue could be considered in caring for patients with chronic chikungunya disease.

## Introduction

Chronic fatiguing illnesses following well-documented infections and acute ‘infectious-like’ illnesses of uncertain cause have been reported for many decades [1]. Myalgic encephalomyelitis/chronic fatigue syndrome (ME/CFS), a neurobiological disease affecting brain functioning, is a classical complication of infection, by the persistence of the pathogen, or persistence of antigenic debris inciting the immune system [2, 3]. Although its aetiology is largely unknown, CFS has been characterised with several persistent or neurovirulent pathogens, such as Epstein Barr virus [4], cytomegalovirus [5], human herpes viruses (HHV6, HHV7) [6], parvovirus B19 [7], enteroviruses [8], *Chlamydia pneumoniae* and *Mycoplasma* spp. [9, 10], *Borrelia burgdorferi* [11], *Brucella melitensis* [12], *Coxiella burnetii* [4]. Ross River virus (RRV), an alphavirus belonging to the arthritogenic Semliki forest virus (SFV) serocomplex, can also trigger a post-infective CFS [4].

Chikungunya virus (CHIKV) is another enveloped RNA positive-strand alphavirus (*Togaviridae* family) of the SFV serocomplex [13]. Chikungunya is a vector-borne disease transmitted by *Aedes* mosquitoes. CHIKV infection is clinically and virologically reminiscent of epidemic polyarthritis caused by the RRV in Australia, or by Sindbis virus-related diseases (referred as Pogosta, Ockelbo or Karelian fever) in Scandinavia (this latter alphavirus belongs to the Western Equine Encephalitis serocomplex) [13]. Thus, the hallmark of CHIKV infection relies on its ability to cause prolonged manifestations such as long-lasting incapacitating rheumatic musculoskeletal pain (LRMSP), mostly arthralgia or even arthritis that the experts henceforth define as post-chikungunya rheumatic disorder [14–20]. Of note, CHIKV can also trigger or even reveal definitive inflammatory rheumatic diseases (IRD) [21–23]. Beyond this classical picture, CHIKV is also associated with a broad spectrum of other early-onset persisting, or late-onset relapsing or lingering manifestations, such as neuropsychological, sensorineural, mood disorders, impairing the quality of life [14, 24–26]. Furthermore, a high proportion of CHIKV-infected people during the 2005–2006 outbreak Reunion island reported prolonged fatigue [24, 25], up to 6 years after the onset of the infection [26]. Lastly, CHIKV was found to persist in peri-synovium macrophages and trigger a long-lasting innate immune response [27], together with being shown as a neuropathogen of public health importance [28].

Interestingly, LRMSP in the context of RRV infection are believed to be the consequences of pre-existing comorbidities, rather than to the onset of a chronic IRD [29]. Finally, we identified similarities between the symptoms and the natural history of chikungunya, and those of IRD and post-infective fatigue syndrome [25, 30]. However, the specificity and causal relationship between CHIKV infection and post-infective fatigue syndrome, as defined by stringent consensus criteria, have still to be clarified.

We hypothesised that a significant proportion of LRMSP reported after CHIKV infection (aka post-chikungunya disease or residual symptoms) especially in a population-based study, could be indeed attributable to a post-infective fatigue syndrome or a CFS, rather than to a primarily rheumatic disease. Consistent with this hypothesis, chikungunya was first reported as a rheumatic disease [14–23], which could make its presentation sensitive to observation bias (Hawthorne effect), CHIKV-infected subjects and authors being tempted to give more credit to synovitis by desirability bias or conformism rather than to another pathogenesis.

We therefore carried out a comprehensive epidemiological study to determine if CHIKV infection was associated with chronic fatigue. For this purpose, we compared the disease burden of LRMSP, idiopathic chronic fatigue (ICF) and CFS-like illness according to CHIKV exposure and among CHIKV-infected subjects with respect to the clinical status (symptomatic/asymptomatic) at disease onset. In addition, we sought to identify the prognostic factors of post-infective syndromes and to describe the overlap between LRMSP, ICF and CFS-like illness, among CHIKV-infected subjects.

## Methods

### Design, population and setting

We used data from the population-based TELECHIK cohort study [25]. This follow-up survey was conducted on Reunion island between November 2007 and May 2008, on average 18 months after the end of the 2005–2006 outbreak. The study sampling and sampling regime have been described elsewhere [25, 30]. It involved a random subset of the SEROCHIK serosurvey that revealed that 38.2% of the community of Reunion island (300 000 people) were CHIKV-infected subjects [31]. For the current work, all the 1094 participants enrolled in the cohort (512 seropositive CHIK+ and 582 seronegative CHIK− subjects) were analysed. The main differences in both analyses of the TELECHIK study are listed in Appendix 1.

### Data collection and follow-up

Participants were interviewed by means of a phone inquiry, following a mean time of 16 months from the SEROCHIK survey (range: 13–20 months, on average 15.93 months for CHIK+ *vs.* 15.97 months for CHIK−) [25]. Parents or legal guardians were questioned for the children aged below 15 years old. A short questionnaire was administered by a French and Creole-speaking investigator blinded of CHIKV exposure [25, 30].

The questionnaire consisted of closed questions on the most frequent symptoms: musculoskeletal pain, fatigue, headaches, digestive disorders, fever, sleep disorders, memory troubles, attention difficulties, mood disturbance, feeling of depression, blurred vision, hearing difficulties, skin disorders, alopecia. The date of the phone inquiry was noted. Participants were asked to declare if the symptoms were present: at the onset or the acute phase of CHIKV infection (or at the time of the peak of the outbreak for seronegative subjects) (t0), at the time of the serosurvey *face-to-face* inquiry (t1), in the interval of the two surveys (t2) or at the time of the follow-up by telephone survey (t3). It was thus possible to identify the unique or repeated character of symptoms and to define their patterns of evolution.

To minimise recall and reporting biases, we retained for t1 information the data collected during the serosurvey. A minimum period of at least 1 month prior to the follow-up call was required to distinguish the symptoms present between the two surveys and those ongoing during the last week before the phone inquiry. Participants lost to follow-up were excluded.

### Outcome measures

Given regular criticisms on the lack of sensitivity of ME/CFS definitions [1], we truncated the Centers for Disease Control (CDC)-1994/Fukuda's definition [32] to only four criteria to keep sufficient sensitivity and be discriminative enough among the main chronic pain phenotypes. These criteria were chosen to fulfil a declarative survey (exclusion of lymphadenopathy, the only clinical criterion of the definition). Sore throat, the other declarative Fukuda's criterion was considered too sensitive to influenza cycles for being discriminative. In the absence of physical examination by a clinician or a psychiatrist, and in the absence of routine biological tests, we adapted the definition of ICF and CFS proposed by the CDC to enable the classification of complaints into LRMSP, ICF and CFS-like illness phenotypes.

Our definition of self-reported CFS-like illness fulfilled the definition of post-exertion malaise, taken as a major criterion, as previously required by the successive conceptual frameworks used for ME/CFS [1, 2, 32]. This fatigue must have lasted consecutively for at least 6 months and not be substantially alleviated by rest and result in a substantial reduction in previous levels of occupational, educational, social, or personal activities.

The absence of self-reported pre-existing comorbidity constituted our second major criterion. It was defined in the absence of alcohol and/or other substance abuse (within 2 years before the onset of chronic fatigue and afterwards), diabetes mellitus, hypertension, renal failure, ischaemic heart disease, asthma, chronic obstructive pulmonary disease, obstructive sleep apnoea, cancer, IRD, osteoarthritis, fibromyalgia, HIV or HCV infection or major psychiatric disorder, to be labelled as ‘post-chikungunya’.

#### Long-lasting rheumatic musculoskeletal pain

Muscle, bone or joint pains were classified as LRMSP when they persisted for at least 6 months in the absence of any pre-existing complaints. They were reported either remitting-relapsing, or lingering, whether they were reported at one or two of the three time-points t1, t2 or t3, or reported at each of the three time-points, respectively.

#### Idiopathic chronic fatigue

Chronic fatigue was defined as a post-exertion difficulty or an impossibility to undertake the same activity after minimal physical or mental exertion within a reduced timeframe (e.g., within the same or several days). ICF was thus defined as a self-reported prolonged fatigue, either remitting–relapsing (recurrent episodes of fatigue lasting ⩾1 month) or lingering (persistent) over a minimum of six consecutive months (this being calculated for CHIK+ subjects between the onset of chikungunya and the follow-up survey, and for CHIK− subjects, between the peak of the outbreak and the follow-up survey).

#### CFS-like illness

CFS-like illness was defined in the presence of both two abovementioned major criteria and at least, two of the following minor criteria: musculoskeletal pain, memory troubles/attention difficulties, new type of headaches (occurred after the time elapsed for acute-stage disease and lasting ⩾1 month. in the absence of migraine), sleeping disorders/unrefreshing sleep.

Minor symptoms had to occur concomitantly or after the onset of reported fatigue.

### Statistical analysis

#### Sample size

The TELECHIK population was large enough to enable the detection of a twofold prevalence of chronic fatigue phenotypes among CHIK+ subjects, given an expected baseline prevalence of 10% of self-reported chronic fatigue among CHIK− subjects [33], an alpha risk of 5%, a statistical power of 90%, and an observed ratio of exposed: unexposed of 1:1.1. This could be achieved with 251 and 286 subjects by the group, respectively. With 512 seropositive CHIK+ and 582 seronegative CHIK−, the power of the present study was deemed sufficient.

#### Prevalence ratios (PR) and impact measures

In contrast to our previous analyses [25, 30], we considered the duration of symptoms across the different time-points of follow-up to define LRMSP, ICF, CFS-like illness or recovery. We estimated the prevalence rates of LRMSP, ICF and CFS-like illness by measuring their occurrence within the timeframe between the outbreak and the follow-up survey in the whole TELECHIK population. Proportions of subjects matching the definition of each of the three outcomes were compared between groups using a Wald test weighted on the sampling fraction. PR and 95% confidence intervals (CI) were estimated using modified Poisson regression models for dichotomous outcomes, which ensure a better interpretability of effect measures [34]. Gender, age and comorbidities were controlled in these analyses, as done previously [25].

Population attributable fractions (PAF) were calculated to assess the community burden of chikungunya for each post-infective syndrome at population level, as follows:
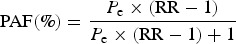
with *P*_e_, the estimated proportion of the population that is exposed to the risk factor (i.e., chikungunya) and RR, the relative risk estimate for the risk factor of interest (RR ⩾1).

Etiologic fractions (EF) among the exposed were estimated in CHIKV-infected subjects to account for the contribution of CHIKV infection in the occurrence of each chronic pain phenotype. They were calculated as follows:
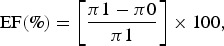
with *π*1: prevalence of the symptom among CHIK+ subjects and *π*0: prevalence of the symptom among CHIK− subjects [25].

#### Contributions of LRMSP, ICF or CFS-like illness to post-chikungunya disease

Because of possible interactions between these outcomes, we explored the spectrum of the three different phenotypes in CHIKV-infected subjects who presented LRMSP or fatigue at disease onset (t0). Then we identified clinical profiles of each phenotype using data from each time-point of follow-up. For this purpose, we performed multiple correspondence (MCA) followed by hierarchical ascendant clustering (HAC) analysis to identify the clusters of symptom characteristics of each phenotype. Multidimensional scaling (MDS) analysis allowed us to confirm the relevance of prognostic factors identified with the abovementioned models. Finally, we constructed a Venn diagram to display the relative contributions of each phenotype of interest to post-chikungunya disease with their respective overlaps.

#### Prognostic factors

We sought to identify the factors associated with LRMSP, ICF and CFS-like illness in adult CHIKV-infected subjects (⩾15 years) who presented LRMSP or fatigue at t0 using multinomial logistic regression models, as done previously for LRMSP [30]. Thus, we tested the assumption that the extent of symptoms at the onset of infection and magnitude of CHIKV-specific IgG response at the time of serosurvey could be also associated with chronic fatigue.

All the interaction terms between the variables included in the models were tested using the Mantel–Haënszel method [35]. Individuals with the above-mentioned comorbidities were excluded from these analyses to allow better interpretability of prognostic factors for long-term outcomes.

#### Sensitivity analyses

Because the association between CHIKV infection and CFS-like illness could be due to other unobserved factors, we checked the robustness of the crude PR and PAF of chikungunya after matching cases of CFS-illness on a propensity score. This propensity scale was built-up in scoring the distance of each post-infective symptom on the dendograms of HAC analysis illustrative of the predictive value of symptoms at the different time-points (t1, t2, t3) for chronic fatigue over the follow-up (Appendix 2). The propensity score was matched together with gender, age, comorbidities and the duration of follow-up, using the Mahalanobis distance method [36]. A ratio of one case for one to four controls was deemed sufficient to run a conditional logistic regression model adjusted on the above-mentioned confounders [25].

For all these analyses, the sampling plan was taken into account [25, 30, 31]. These were performed using Stata14^®^ (StataCorp. 2015, College Station, USA) excluding the observations with missing data. Statistical significance was set at *P* = 0.05.

## Ethics and funding

The SEROCHIK serosurvey had received approval from the ethical committee for studies with human subjects of Bordeaux (No 2006/47) and from the National Commission for Informatics and Liberty. It was funded by the National Institute of Health and Medical Research (INSERM). Participants had previously given their written consent and were asked to provide oral consent for the follow-up study. Participants and INSERM were neither involved in the study design, conduction nor in the definition of outcomes. The study respected the STROBE statement (Appendix 3).

## Results

**Fig. 1.**
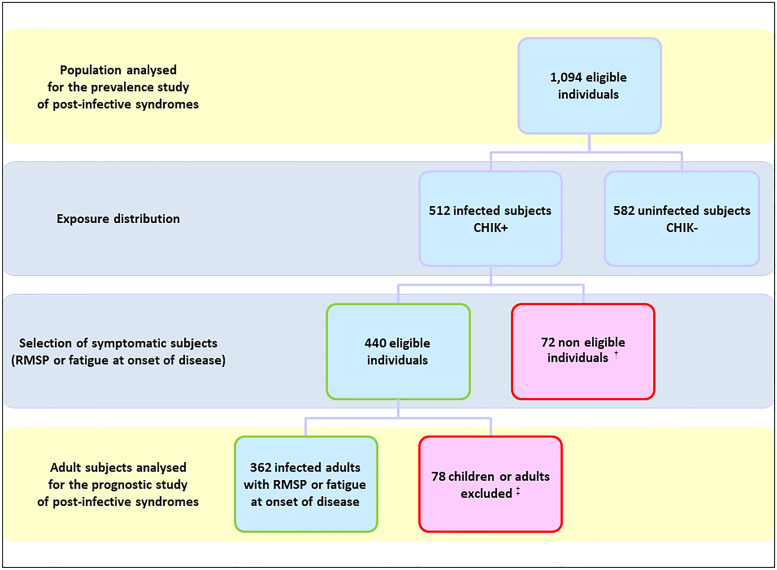
Flowchart of the population, TELECHIK cohort study, Reunion island, November 2007–May 2008. ^†^64 asymptomatic subjects and eight symptomatic subjects presenting neither rheumatic musculoskeletal pain nor fatigue at the onset of disease. ^‡^50 children, 15 adults with absent contact or relocated, 13 subjects with absence of overt temporality in the clinical course of the post-chikungunya disease.

The study populations were based on the TELECHIK cohort survey [25, 30]. The selection process for the reanalysis is detailed in Figure 1 and its background selection from the SEROCHIK population is displayed in the Figure S1.

### Socio-demographic characteristics

The descriptive characteristics of the study populations are presented in Table 1. The populations were shifted towards an overrepresentation of women (>57% *vs.* 51% in census data) and of middle-aged subjects (30–59 years, >49% *vs.* 40% thereof), consistent with the assumption that CHIKV was transmitted in the home environment, to impact the most regular residents.

**Table 1.** Characteristics of the population analysed for the prevalence and the impact of post-infective syndromes at population level and of the sample analysed for the prognostic study of post-infective syndromes, TELECHIK cohort study, Reunion island, 2006–2008

Population	Entire cohort	Prognostic study
Total	1094	362
Time of follow-up, mean (range)	23 months (19–34 months)^a^	24 months (15–36 months)^b^
Characteristics, *n* (%)		
Gender		
Women	645 (57.4)	224 (61.9)
Men	449 (42.6)	138 (38.1)
Age		
<15 years	139 (12.7)	–
15–29 years	199 (18.2)	66 (18.2)
30–44 years	244 (22.3)	94 (26.0)
45–59 years	282 (25.8)	98 (27.1)
⩾60 years	230 (21.0)	104 (28.7)
Body mass index		
<25.0 kg/m^2^	686 (64.2)	195 (54.2)
25–29.9 kg/m^2^	298 (27.9)	121 (33.6)
⩾30 kg/m^2^	85 (7.9)	44 (12.2)
Pre-existing comorbidities		
None	727 (66.5)	–
One or more	367 (33.5)	–

a Since peak of the outbreak (February 15, 2006), on average 24 months (range 20–34 months) for CHIK+ subjects and 23 months (range 21–34 months) for CHIK− subjects.

b Since onset of infection. Data are given as numbers and unweighted percentages in parentheses.

### PRs at population level and in CHIKV-infected subjects

The prevalence rates and crude PR associated with LRMSP, ICF and CFS-like illness are reported at the population level and among CHIK+ subjects in Table 2.

**Table 2.** Prevalence and crude prevalence ratios for post-infective syndromes, at population level and among chikungunya virus-infected subjects at onset of infection, TELECHIK cohort study, Reunion island, 2006–2008

Population level				
Outcome^a^	CHIK− (%)	CHIK+ (%)	Crude PR (95% CI)	*P* value
LRMSP	8.9	29.9	3.4 (2.4–4.6)	<0.001
ICF	12.7	34.3	2.7 (2.0–3.6)	<0.001
CFS-like illness	6.7	21.5	3.2 (2.1–4.7)	<0.001
Chikungunya-virus-infected subjects				
Outcome^a^	Asymptomatic (%)	Symptomatic (%)	Crude PR (95% CI) (%)	*P* value
LRMSP	8.7	32.9	3.7 (1.5–9.0)	0.003
ICF	8.5	38.7	4.5 (2.0–9.9)	<0.001
CFS-like illness	7.4	23.9	3.2 (1.4–7.5)	<0.001

LRMSP, long-lasting rheumatic musculoskeletal pain; ICF, idiopathic chronic fatigue; CFS, chronic fatigue syndrome.

Prevalence rates are weighted on the sampling fraction and prevalence ratios (PR) are given with 95% confidence intervals (95% CI).

a Each outcome was identified as persistent, remittent-relapsing or lingering, between its first occurrence at the onset of infection and the week before the TELECHIK survey.

Baseline-proxy prevalence rates of the three chronic pain phenotypes in the community could be determined in CHIK− subjects, taken as a proxy of the general population. They were of 8.9%, 12.7% and 6.7%, for LRMSP, ICF and CFS-like illness, respectively.

Interestingly, compared to our initial analysis considering a single cross-sectional outcome [25], the fact of using the full data collection over the follow-up and the chronology and the duration of symptoms in the definition of outcomes revealed that each chronic pain phenotype was strongly associated with CHIKV exposure, both at population level and among CHIKV-infected subjects.

### Population attributable and etiologic fractions

At the population level, LRMSP, ICF and CFS-like illness were highly attributable to chikungunya with PAFs as 43.2% for LRMSP, 36.2% for ICF and 41.0% for CFS-like illness (Table 3).

**Table 3.** Population attributable and etiologic fractions of chikungunya for post-infective syndromes, at population level and among chikungunya-virus-infected subjects, TELECHIK cohort study, Reunion island, 2006–2008

Population level	
Outcome^a^	PAF (95% CI) (%)
LRMSP	43.2 (34.1–51.1)
ICF	36.2 (27.7–43.7)
CFS-like illness	41.0 (29.9–50.3)
Chikungunya virus-infected subjects	
Outcome^a^	EF (95% CI) (%)
LRMSP^b^	70.3 (61.7–79.0)
ICF^c^	63.0 (53.9–72.0)
CFS-like illness^c^	68.6 (57.8–79.4)

LRMSP, long-lasting rheumatic musculoskeletal pain (LRMSP); ICF, idiopathic chronic fatigue (ICF); CFS, chronic fatigue syndrome.

Population attributable fractions (PAF) are estimated for adjusted predictors on gender, age and comorbidities.

a Each outcome was identified as persistent, remittent-relapsing or lingering, between its first occurrence at the onset of infection and the week before the TELECHIK survey.

b Comorbidities controlled are osteoarthritis or other.

c Comorbidities controlled are diabetes mellitus, hypertension, ischemic heart disease, asthma, chronic obstructive bronchopulmonary disease, renal failure, cancer. Crude etiologic fractions (EF) are given with 95% confidence intervals (95% CI).

In CHIKV-infected subjects, the three phenotypes could be attributable to CHIKV infection in even higher proportions, as evidenced by EF ranging between 63.0% for ICF and 70.3% for LRMSP, the one for CFS-like illness, the phenotype of interest, being as high as 68.6%.

### LRMSP, ICF and CFS-like illness in MDS

The analysis of the clinical profiles of LRMSP, ICF and CFS-like illness using MCA and HAC confirmed the clustering of each symptom at the different time-points. Importantly, MDS failed to identify a definite pattern associated with LRMSP or ICF. The symptoms associated with both phenotypes were very similar whichever the time-point (data not shown). Interestingly, at t1, the symptoms associated with LRMSP and ICF belonged all to the spectrum of CFS-like illness with exception of blurred vision (Fig. S2). Interestingly, hearing difficulties, mood disturbance and feeling of depression that do not belong to the spectrum of CFS-like illness were not clustered with fatigue symptoms (Fig. S3).

### Contribution of LRMSP, ICF and CFS-like illness to post-chikungunya chronic disease

**Fig. 2.**
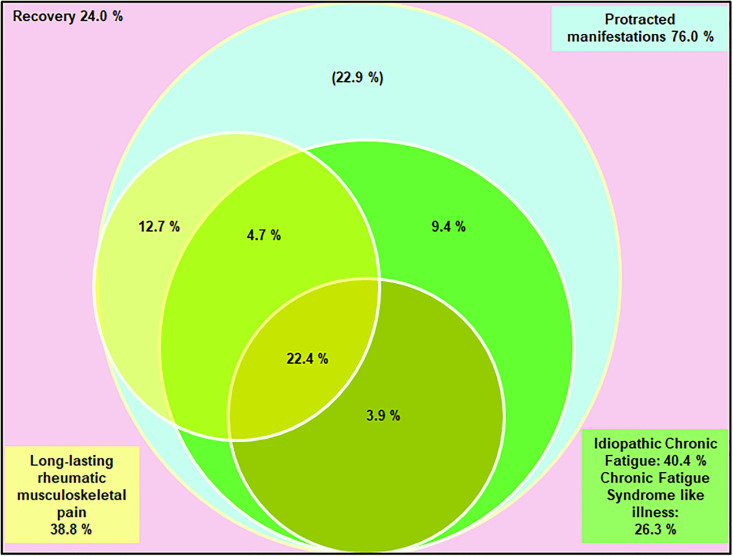
Relationships between the three main post-chikungunya syndromes reported among infected subjects aged 15 years and over who declared symptoms at the onset of infection, TELECHIK cohort study, Reunion island, November 2007–May 2008. The percentage in the left upper corner (light pink) accounts for the subjects who recovered. The percentage in the right upper corner (light blue frame) accounts for the ensemble of all long-lasting post-chikungunya disease manifestations. Percentages into the Venn diagram accounts for the proportions of the three long-lasting post-infective syndromes of interest: long-lasting rheumatic musculoskeletal pain (yellow circle and left bottom frame), idiopathic chronic fatigue (green light circle and right bottom frame) and chronic fatigue syndrome-like illness (dark green bottom circle). In light blue are other manifestations remaining to be specified, as sleep disorders (*n* = 23), memory troubles (*n* = 20), blurred vision (*n* = 15), depression (*n* = 12), attention difficulties (*n* = 6), hearing difficulties (*n* = 4) and mood disturbance (*n* = 3).

The contribution of each phenotype, defined as clusters of persistent symptoms on follow-up, to post-chikungunya chronic disease was modelled in the Venn diagram (Fig. 2). Prolonged manifestations were present in 76% of CHIK+ subjects. LRMSP and ICF (with or without CFS-like illness) accounted for 53.1% of these manifestations. LRMSP and ICF coexisted in more than 25% of initially symptomatic subjects. LRMSP and CFS-like illness coexisted in more than 20% of these subjects. Of note, ICF was the most commonly reported phenotype.

### Prognostic factors of LRMSP, ICF and CFS-like illness

The factors associated with LRMSP, ICF and CFS-like illness throughout the follow-up of CHIKV-infected subjects are summarised in Tables S4–S6, respectively.

Female gender was associated both with more persistent fatigue and features of CFS-like illness than LRMSP. Age ⩾60 years was associated with each of the three post-infective syndromes. Extensive acute stage, a proxy of the severity of acute chikungunya illness, was associated with lingering LRMSP, lingering ICF and CFS-like illness. Post-epidemic CHIKV-specific IgG level increased the likelihood for lingering LRMSP but was protective for remitting-relapsing fatigue. Taken together, these results identified female gender as predictive of chronic fatigue and the humoral immune response as predictive of rheumatic pain, while age and extensive acute stage were indicative of both pathogeneses. Extensive acute stage illness was not associated with a hyperimmune response (data not shown).

### Sensitivity analyses

The distribution of the matching criteria among case and controls are displayed in Table S7. Interestingly, the PAF value of chikungunya for CFS-like illness obtained using the propensity score's matching was slightly decreased from 41.0% to 33.3% (Table 3 and Table S8).

## Discussion

Here, we report a reanalysis of the TELECHIK survey, a large cohort study dealing with the community burden of post-chikungunya chronic disease on Reunion island [25].

The new findings confirm the high disease burden of post-infective phenotypes, both at the population level and in CHIKV-infected subjects, on average 18 months after the end of the epidemic. The absence of previous herd immunity to CHIKV was a key determinant for the magnitude of this outbreak, with about 40% of the community infected with the virus [31]. This added probably to the high prevalence of LRMSP, ICF and CFS-like illness among CHIK+ subjects 2 years after the onset of infection, which may account for the heavy toll paid by the Reunionese population. From this point of view, the public health impact of CHIKV in Reunion island was far superior to that reported for RRV-related epidemic polyarthritis in Australia, whose spectrum of long-term manifestations is very similar [4, 29]. Consistent with this observation is the long-lasting deterioration of the quality of life with chikungunya [24, 26], whereas post-infective syndromes (LRMSP or fatigue phenotypes) in the setting of Sindbis-virus-related diseases [37] or RRV infection [4] are more rapidly reversible and affect less permanently the daily activities, as the putative consequences of comorbidities with RRV [29].

However, the breakthrough of our reanalysis relies on the evidence that when more rigorously defined, based on the duration of symptoms, the persistent manifestations of chikungunya belong as much to the spectrum of chronic fatigue than that of a primarily rheumatologic disease. This underscores the fact that unlike most studies reported so far [14, 15, 20–23], largely conducted within hospital wards [14, 22], or in patients seeking care in primary healthcare centres [15] or rheumatology clinics [20–23], our cohort study was population-based and therefore less prone to be skewed by a selection bias towards the most severe pathogenesis, as observed in the above-mentioned situations, including severe arthritis. In agreement with, or in accordance, the prominent role played by undifferentiated inflammatory musculoskeletal pains in population-based studies [16, 17]. This may represent up to 95% of post-chikungunya chronic pains [20], which gives room for alternative diagnoses to rheumatic disease.

Prolonged fatigue has been increasingly recognised as a companion symptom of post-chikungunya rheumatic disorders [15, 18, 19, 24–26]. ME/CFS is classified by the World Health Organization as a disease of the central nervous system, whose neuropathogenesis shares many aspects with that of encephalomyelitis disseminata/multiple sclerosis, including grey matter volume reduction, white matter hyperintensities, astrogliosis with astrocyte dysfunction, neuroinflammation, central hypometabolism, brainstem hypoperfusion, oxidative and nitrosative reactive stress, autoantibodies and mitochondrial defects [3].

Here we show that the full spectrum of chronic fatigue is common and involved in 40% of post-chikungunya chronic disease and that CFS-like illness may account for 26% of these manifestations. These findings are coherent with the studies supporting the neurovirulence of CHIKV [28, 38–41]. Indeed, CHIKV infection has been associated clinically with Guillain–Barré syndrome [38], neuropathic pain [39], encephalitis [28], and experimentally, with neuropathological evidence of neuron and astrocyte invasion, astrogliosis and engagement of a multifaceted innate immune response from astrocytes, microglia and resident dendritic cells [40, 41]. The latter findings are consistent with the recent report of a fatal form of Q-Fever fatigue syndrome, linking astrogliosis with the invasion of astrocytes [42], which is also compatible with the abovementioned pathogenesis proposed for ME/CFS [3]. Altogether, these observations fuel the assumption that CHIKV, like *C.burnetii* [42], could be a pathogen able to evade the host immune response, shelter and replicate in host sanctuaries [27]. Whether an encephalitic event is a prerequisite for the putative neuro-invasiveness of CHIKV or that mild encephalitis could trigger the CFS-like illness found in chikungunya chronic patients remains to be shown but may reopen the debate on the infectious cause of CFS [2]. Interestingly, the spectrum of musculoskeletal pain, assembled in LRMSP, ICF and CFS-like illness phenotypes accounted only for 53.1% of all prolonged manifestations, the remaining 22.9% belonging to the spectrum of mood disorders (e.g., anxiety or depression), neurobehavioral disorders or sensorineural disorders. This latter finding fuels the assumption that persistent post-chikungunya chronic disease may be also attributable to other conditions, as previously proposed for RRV [29].

Several studies have identified predictors for LRMSP in CHIKV-infected subjects [18, 19, 30]. Our reanalysis of prognostic factors, based on outcomes defined with more accuracy on the duration of manifestations, contrasts with the previous findings, while allowing two distinct patterns for LRMSP and chronic fatigue phenotypes to be distinguished. Thus, the most persistent form of post-chikungunya rheumatic disorder was associated with age ⩾45 years, extensive acute stage (i.e., polyarthralgia with general symptoms), and enhanced host adaptive immune response, while the most persistent forms of post-infective fatigue were associated with female gender, extensive acute stage, or age ⩾60 years. Between the three extreme phenotypes, remittent-relapsing outcomes could also be associated to a lesser extent to these prognostic factors. The sex difference in the burden of chronic fatigue has been observed for a long time in population-based studies with women paying the heaviest toll [43–45]. Among the factors susceptible to explain this gender disadvantage in the context of epidemiologic transition on Reunion island, psychosocial stress resulting from intimate partner violence, poor mental health, or early repeated pregnancies seem to us particularly obvious [45–49]. Importantly, the more extensive acute stage was predictive of CFS-like illness, which supports the early hypothesis that chronic symptoms of alphaviral arthritogenic infections are driven by the severity of the acute illness [4]. Interestingly, we found arguments to suggest this hypothesis was not driven by a hyperimmune mediated humoral response, unlike LRMSP.

Strengths and limitations of the TELECHIK study have been reported previously [25, 30]. In this paper, we emphasised on the likelihood of an observation bias and the possibility that CHIKV-infected subjects or doctors may have reported more symptoms by social desirability bias despite the blinded investigation. In line with this hypothesis, each chronic manifestation assessed in the survey were more commonly reported in CHIK+ than in CHIK− subjects [25]. However, given its ordinariness and the small discrepancy between CHIK− and CHIK+ in our seminal analysis, fatigue was putatively the manifestation the least prone to the Hawthorne effect [25]. Given the more stringent definitions used in our reanalysis and the prevalence of LRMSP, ICF and CFS-like illness in the reference group of CHIK− subjects, in the expected range for self-reports, we strongly believe that those biases are unlikely to have changed the overall sense of our results.

In the context of advances in the understanding of the pathogenesis of the post-chikungunya disease, several lessons can be drawn from our reanalysis of the TELECHIK cohort study. First, given the abovementioned Hawthorne effect, it is possible that previous reports may have slightly overestimated the prevalence of prolonged manifestations of the post-chikungunya disease. Second, using stringent definitions of phenotypes, multidimensional and propensity score analyses, we demonstrated that self-reported post-chikungunya disease is a knot of rheumatic disease, chronic fatigue, mood, neurobehavioral and sensorineural disorders.

In this framework, given the strengths and limitations of our study, we propose that chikungunya patients fulfilling our definition of CFS-like illness be examined clinically by specialists to see whether they fit the diagnosis of CFS. If this is confirmed, we suggest that post-infective fatigue and musculoskeletal disorders be considered as minor phenotypes whereas CFS and rheumatoid-like arthritis be considered as possible extreme phenotypes of post-chikungunya disease, at the bottom or on the top of a likely complex disease, respectively. Further studies are needed to assess the heritability and better understand the pathogenesis of this puzzling problem, as classically proposed for complex diseases.

## Supplementary material

For supplementary material accompanying this paper visit https://doi.org/10.1017/S0950268818000031. click here to view supplementary material
