# Supplementary material for: Rheumatism and chronic fatigue, the two facets of post-chikungunya disease: the TELECHIK cohort study on Reunion island
Source: Epidemiol Infect. 2018 Feb 28;146(5):633–41. doi: 10.1017/S0950268818000031 (PMC5892425; doi:10.1017/S0950268818000031)
Supplement: Supplementary file 1 [file S0950268818000031sup001.doc]

**Supplementary material (online publication only)**

**Supplemental file 1**

**
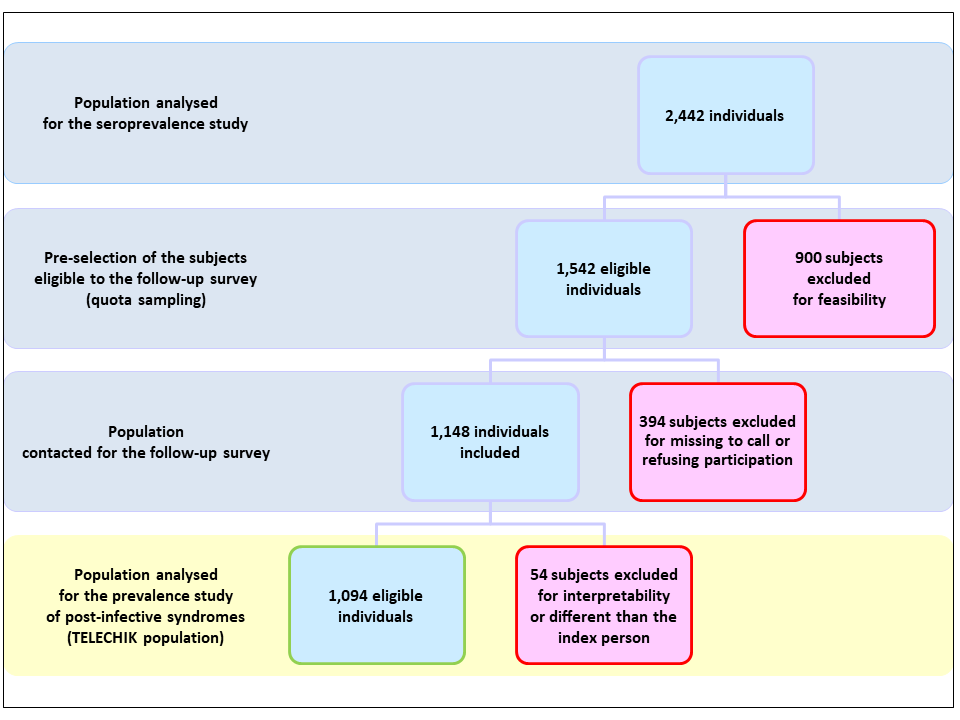
**

**Figure S1. Selection of the TELECHIK population from the SEROCHIK population**

**Supplemental file 2**


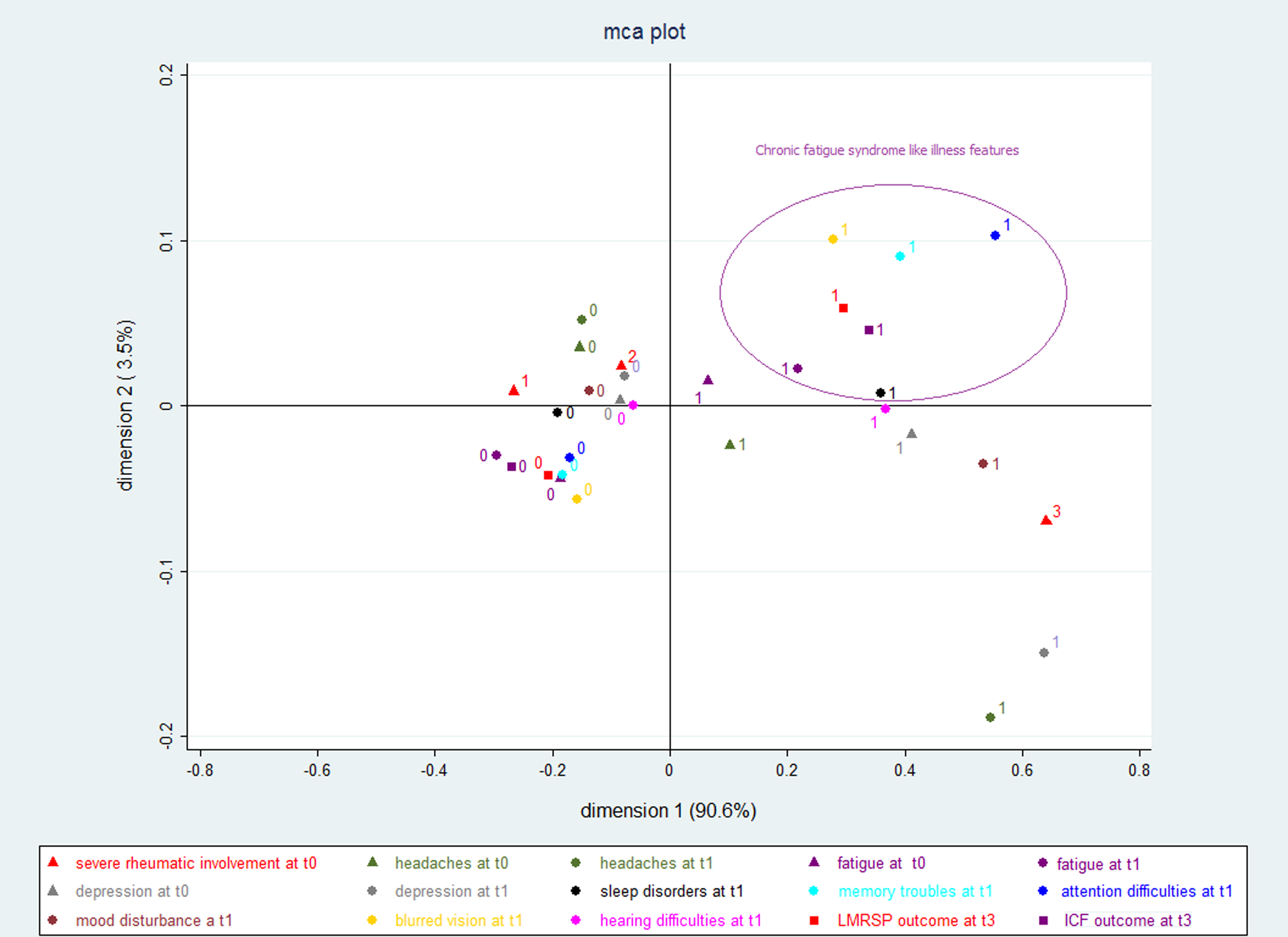


**Figure S2. Multiple correspondence analysis plot of chikungunya manifestations reported at t0 (onset of infection) and t1 (date of serosurvey), TELECHIK cohort study, Reunion island, 2006–2008.**

Symptoms are considered as post-chikungunya disease manifestations and contribute to the inertia of the cloud of points whose dimension 1 explains 90.6% of the total inertia. The two outcomes are projected as passive supplementary variables (they do not contribute to inertia). Unless for the less reported symptoms, the farther is a point from the axis, the stronger is its contribution to the dimension. Interpretation: Except for blurred vision, the circle and the right upper quadrant assemble symptoms which belong to the spectrum of chronic fatigue syndrome like illness. Rheumatic musculoskeletal pain at t1 and the symptoms at t2 were not entered in the model, because the former identified 100% of long-lasting rheumatic musculoskeletal pains, and the latter entered in the definition of the outcomes.

**Supplemental file 3**


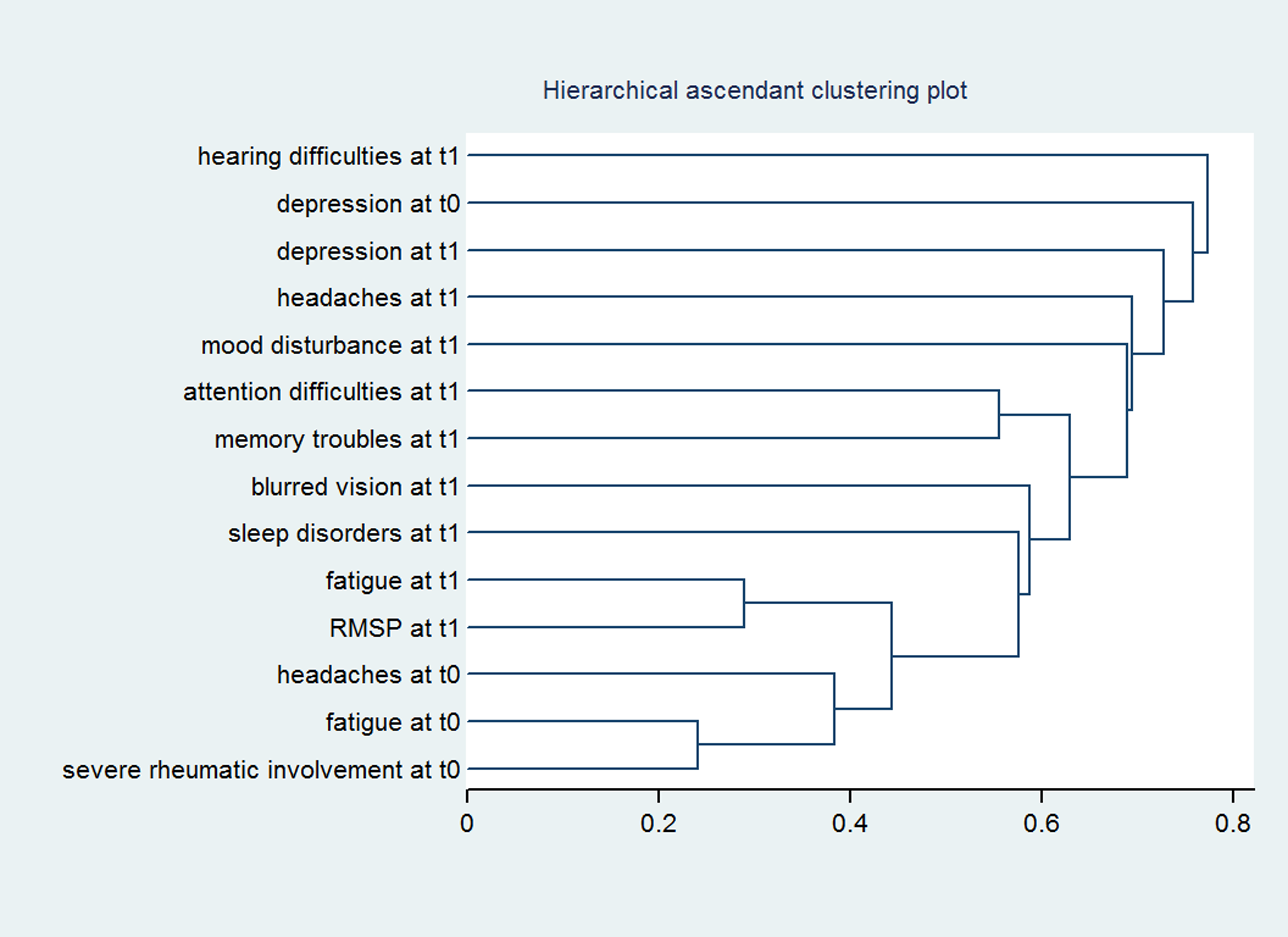


**Figure S3. Hierarchical ascendant clustering plot of the chikungunya manifestations reported at t0 (onset of infection) and at t1 (date of serosurvey), TELECHIK cohort study, Reunion island, 2006–2008.**

The symptoms reported at onset of infection or at the date of the serosurvey are plotted in hierarchical ascending order according to the strength of the linkage, using a single-linkage clustering method. The closer is the symptom from the bottom axis, the stronger is the linkage with chronic fatigue. The farther is the symptom from the bottom axis, the weaker is the linkage with chronic fatigue.

| **Supplemental file 4**  **Table S4. Adjusted odds ratio (OR) for long-lasting rheumatic musculoskeletal pain (LRMSP) prognostic factors among the initially symptomatic CHIKV-infected subjects all through the TELECHIK cohort study follow-up (t3-t0), Reunion island, 2006-2008** | | | | | | |
| --- | --- | --- | --- | --- | --- | --- |
| **Outcome** | **Remittent-relapsing LRMSP** | | | **Lingering RMSP** | | |
| **Prognostic factors** | **Adjusted OR** | **95% CI** | ***P* value** | **Adjusted OR** | **95% CI** | ***P* value** |
| **Gender** |  |  |  |  |  |  |
| Male | 1 |  |  | 1 |  |  |
| Female | 1.9 | 0.6 – 5.4 | 0.217 | 1.3 | 0.7 – 2.3 | 0.346 |
| **Age (years)** |  |  |  |  |  |  |
| 15 to 29 | 1 |  |  | 1 |  |  |
| 30 to 44 | 4.0 | 0.8 – 17.9 | 0.074 | 2.3 | 0.8 – 6.3 | 0.117 |
| 45 to 59 | 2.8 | 0.5 – 13.3 | 0.202 | 5.1 | 1.9 – 13.7 | 0.001 |
| ≥ 60 | 7.4 | 1.5 – 35.6 | 0.012 | 10.1 | 3.7 – 27.5 | < 0.001 |
| **Symptoms extent at chikungunya acute stage** |  |  |  |  |  |  |
| Mild to moderate ^†^ | 1 |  |  | 1 |  |  |
| High ^‡^ | 1.2 | 0.3 - 4.2 | 0.759 | 3.0 | 1.4 - 6.5 | 0.004 |
| **Specific anti-CHIKV IgG titer** (for one optical density unit increase) | 1.4 | 0.6 - 2.9 | 0.372 | 1.7 | 1.1 - 2.5 | < 0.001 |
| OR: odds ratio; 95% CI: 95% confidence interval. Estimates are weighted on the sampling fraction.  ^†^ Musculoskeletal pain with at least two of the following conditions: fever, ≥ 6 painful joints or ≥ 4 other symptoms among: fatigue, headache, depression, rash. ^‡^ Musculoskeletal pain with all of the three following conditions: fever, ≥ 6 painful joints or ≥ 4 other symptoms | | | | | | |
| **Supplemental file 5**  **Table S5. Odds ratio (OR) for idiopathic chronic fatigue (ICF) prognostic factors among the initially symptomatic CHIKV-infected subjects all through the TELECHIK cohort study follow-up (t3-t0), Reunion island, 2006-2008** | | | | | | |
| **Outcome** | **Remittent-relapsing ICF** | | | **Lingering ICF** | | |
| **Prognostic factors** | **Adjusted OR** | **95% CI** | ***P* value** | **Adjusted OR** | **95% CI** | ***P* value** |
| **Gender** |  |  |  |  |  |  |
| Male | 1 |  |  | 1 |  |  |
| Female | 1.1 | 0.4 – 2.8 | 0.872 | 1.9 | 1.1 – 3.2 | 0.030 |
| **Age (years)** |  |  |  |  |  |  |
| 15 to 29 | 1 |  |  | 1 |  |  |
| 30 to 44 | 1.5 | 0.3 – 6.2 | 0.604 | 2.2 | 0.8 – 5.3 | 0.086 |
| 45 to 59 | 1.5 | 0.3 – 6.1 | 0.559 | 2.4 | 0.9 – 5.8 | 0.063 |
| ≥ 60 | 0.8 | 0.1 – 4.2 | 0.765 | 5.5 | 2.3 – 13.1 | < 0.001 |
| **Symptoms extent at chikungunya acute stage** |  |  |  |  |  |  |
| Mild to moderate ^†^ | 1 |  |  | 1 |  |  |
| High ^‡^ | 5.0 | 1.6 - 14.9 | 0.004 | 4.5 | 2.1 - 9.3 | < 0.001 |
| **Specific anti-CHIKV IgG titer** (for one optical density unit increase) | 0.5 | 0.2 - 0.9 | 0.028 | 0.8 | 0.5 - 1.1 | 0.164 |
| OR: odds ratio; 95% CI: 95% confidence interval. Estimates are weighted on the sampling fraction.  ^†^ Musculoskeletal pain with at least two of the following conditions: fever, ≥ 6 painful joints or ≥ 4 other symptoms among: fatigue, headache, depression, rash. ^‡^ Musculoskeletal pain with all of the three following conditions: fever, ≥ 6 painful joints or ≥ 4 other symptoms. | | | | | | |
| **Supplemental file 6**  **Table S6. Odds ratio (OR) for idiopathic chronic fatigue (ICF) and chronic fatigue syndrome (CFS) like illness prognostic factors among the initially symptomatic CHIKV-infected subjects, TELECHIK cohort study follow-up (t3-t0), Reunion island, 2006-2008** | | | | | | |
| **Outcome** | **ICF**  **(remittent-relapsing or lingering)** | | | **CFS-like illness**  **(remittent-relapsing or lingering)** | | |
| **Prognostic factors** | **Adjusted OR** | **95% CI** | ***P* value** | **Adjusted OR** | **95% CI** | ***P* value** |
| **Gender** |  |  |  |  |  |  |
| Male | 1 |  |  | 1 |  |  |
| Female | 1.2 | 0.5 – 2.3 | 0.675 | 2.2 | 1.2 – 4.2 | 0.012 |
| **Age (years)** |  |  |  |  |  |  |
| 15 to 29 | 1 |  |  | 1 |  |  |
| 30 to 44 | 1.5 | 0.5 – 4.5 | 0.423 | 2.5 | 0.8 – 7.1 | 0.084 |
| 45 to 59 | 1.6 | 0.5 – 4.9 | 0.363 | 2.7 | 0.9 – 7.4 | 0.059 |
| ≥ 60 | 2.9 | 1.0 – 8.7 | 0.054 | 5.8 | 2.1 – 15.9 | 0.001 |
| **Symptoms extent at chikungunya acute stage** |  |  |  |  |  |  |
| Mild to moderate ^†^ | 1 |  |  | 1 |  |  |
| High ^‡^ | 2.7 | 1.0 - 7.1 | 0.051 | 6.3 | 3.0 - 12.9 | < 0.001 |
| **Specific anti-CHIKV IgG titer** (for one optical density unit increase) | 0.7 | 0.4 - 1.1 | 0.112 | 0.8 | 0.5 - 1.1 | 0.153 |
| OR: odds ratio; 95% CI: 95% confidence interval. Estimations are weighted according to sampling fractions.  ^†^ Musculoskeletal pain with at least two of the following conditions: fever, ≥ 6 painful joints or ≥ 4 other symptoms among: fatigue, headache, depression, rash. ^‡^ Musculoskeletal pain with all of the three following conditions: fever, ≥ 6 painful joints or ≥ 4 other symptoms. | | | | | | |

**Supplemental file 7**

| **Table S7. Distribution among cases of CFS-like illness and controls of the matching criteria at population level, TELECHIK cohort study, Reunion island, 2006-2008** | | | | | |
| --- | --- | --- | --- | --- | --- |
| **Population level** | **Case** | | **Control** | |  |
| **Matching criteria** | **(n=153)** | | **(n=406)** | | ***P* value** |
| **Female gender, n (%)** | 118 | (77.1 %) | 282 | (69.5 %) | 0.073 |
| **Age, n (%)** |  |  |  |  | 0.493 |
| < 15 years | 5 | (3.3 %) | 12 | (2.9 %) |  |
| 15 to 29 years | 12 | (7.8 %) | 49 | (12.1 %) |  |
| 30 to 44 years | 29 | (18.8 %) | 78 | (19.2 %) |  |
| 45 to 59 years | 53 | (34.6 %) | 149 | (36.7 %) |  |
| **Body mass index, n (%)** |  |  |  |  | 0.957 |
| < 25.0 kg/m^2^ | 84 | (55.6 %) | 221 | (55.1 %) |  |
| 25 to 29.9 kg/m^2^ | 51 | (33.8 %) | 140 | (34.9 %) |  |
| | ≥ 30 kg/m^2^ |  | | --- | --- | | 16 | (10.6 %) | 40 | (10.0 %) |  |
| **Comorbidities, n (%) ^‡^** |  |  |  |  | 0.002 |
| None | 68 | (45.6 %) | 240 | (60.3 %) |  |
| One or more | 81 | (55.4 %) | 158 | (39.7 %) |  |
| **Mean follow-up (± SD), months** | 24.6 | (± 3.7) | 23.7 | (± 2.7) | 0.017 |
| **Mean propensity score (± SD)** | 27.1 | (± 25.4) | 25.5 | (± 22.7) | 0.817 |
| **^‡^** Comorbidities controlled are diabetes mellitus, hypertension, ischemic heart disease, asthma, chronic obstructive bronchopulmonary disease, renal failure, cancer | | | | | |

**Supplemental file 8**

| **Table S8. Prevalence ratios and population attributable fractions of chikungunya for CFS-like illness, estimated from the odds ratios of a conditional logistic regression model matched for the propensity score, TELECHIK cohort study, Reunion island, 2006-2008** | | | |  |
| --- | --- | --- | --- | --- |
| **Outcome CFS-like illness** | **Effect measure** | **(95% CI)** | |  |
| **Crude OR** | 2.5 | (1.6 - 3.9) | |  |
| **Estimated crude PR** | 1.9 | (1.4 - 2.4) | |  |
| **Adjusted OR ^‡^** | 2.7 | (1.7 - 4.3) | |  |
| **Estimated adjusted PR** | 2.0 | (1.5 - 2.5) | |  |
| **Estimated PAF ^‡^** | 33.3 % | (22.1 % - 42.8 %) | |  |
| *CFS: chronic fatigue syndrome. Conditional logistic regression model odds ratios (OR) are obtained after matching for gender, age, body mass index, comorbidities, time of follow-up and a propensity score predictive for CFS-like illness. Prevalence ratios (PR) are estimated from the conditional logistic regression model OR using the method of Zhang and Yu (Zhang J and Yu KF. What’s relative risk? A method of correcting the odds ratio in cohort studies of common outcomes. JAMA 1998; 280: 1690-1). Population attributable fractions (PAF) are estimated from the estimated prevalence ratio adjusted on gender, age and comorbidities.* **^‡^** Comorbidities controlled are diabetes mellitus, hypertension, ischemic heart disease, asthma, chronic obstructive bronchopulmonary disease, renal failure, cancer*.* | | | | |
|  | | |  | |

Supplemental file 9

| **Appendix 1. Main differences between the seminal TELECHIK survey and our reanalysis.** | | | |
| --- | --- | --- | --- |
| **Paper** | **Seminal work (2011, 2013))** | **Re-analysis (2014-2017)** | |
| Population (CHIK+/CHIK-) size | (n=1,094) | (n=1,094) | |
| Number with symptoms of interest at t0 | 346 ^†^ | 362^‡^ | |
| Definition of outcomes  Duration of chronic symptoms | cross-sectional *  unconsidered | longitudinal **  t1-t0 > 6 months, | |
| Course of chronic symptoms | lingering at t1,t2 and t3,  relapsing at t1+ t3, or at t2+t3 | lingering at t1,t2 and t3,  relapsing at t1+ t3, or at t2+t3 | |
| Core statistical analysis | Poisson regression / multinomial logistic regression | Poisson regression / multinomial logistic regression | |
| Sensitivity analysis | Poisson regression | Conditional logistic regression matched on propensity score | |
| CHIK+: infected people diagnosed with a positive chikungunya virus specific IgG serology; CHIK-: uninfected people diagnosed with a negative chikungunya virus specific IgG serology. t0: disease onset, as declared in the SEROCHIK serosurvey (t1). ^†^rheumatic musculoskeletal pain presented at t0; ^‡^ rheumatic musculoskeletal pain or fatigue presented at t0. Symptoms at t1 are reported as still present at the time of the serosurvey. Symptoms at t2 are reported with the interval between the serosurvey and the follow-up survey. Symptoms at t3: are reported within the last week or the day of the follow-up survey | | |  |

**Supplemental file 10**

**Appendix 2. Construction of the propensity score**

The propensity scale is built-up in scoring the distance of each post-infective symptom for each of the three dendograms of hierarchical ascendant clustering (HAC) corresponding to the different time points (t1: time of the serosurvey; t2: interval of the two surveys; t3: time of the follow-up survey) of the follow-up. HAC plots are performed using single shrinkage clustering. These are obtained using Chi2 square distance and Jaccard indices, which measure the dissimilarity between ensembles of binary variables. The closer is the symptom from the bottom axis, the stronger is the linkage with chronic fatigue. Symptoms are then weighed with positive or negative ponderation values according to whether they are compatible or not with the criteria of myalgic encephalomyelitis/chronic fatigue syndrome [i].The ponderation values are displayed onto the branches of each of the three following dendograms. Total propensity score is obtained by summing the subtotals of each time period and ranges between – 20 points and + 100 points.

**Additional references**

1. **Carruthers BM, *et al.*** Myalgic encephalomyelitis : International Consensus Criteria. *Journal of Medicine* 2011; **270**: 327-338.


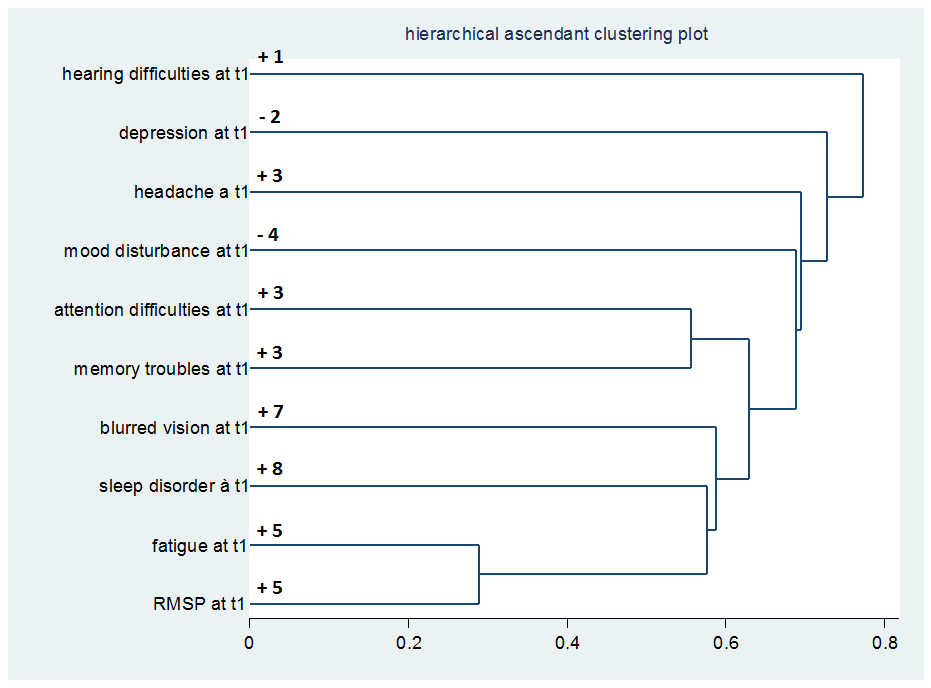


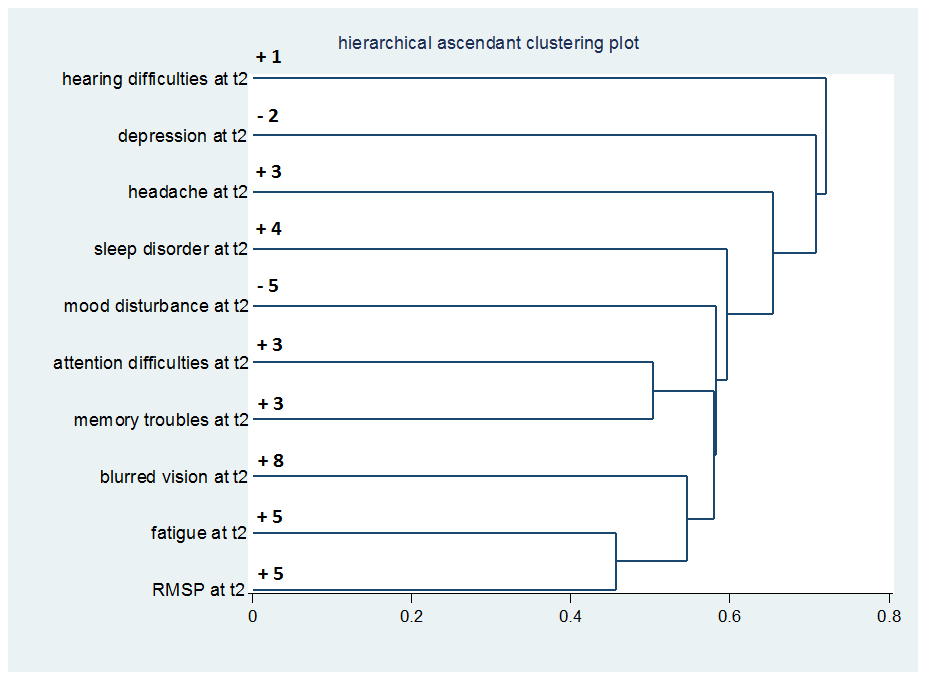


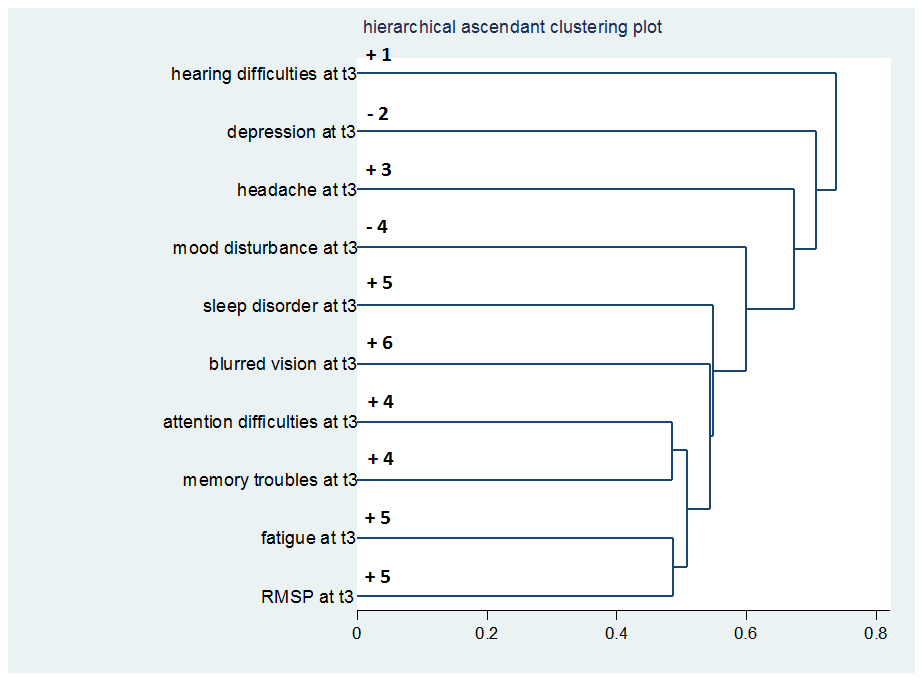


**Supplemental file 11**

***Appendix 3. STROBE checklist of items that should be included in reporting of*** cohort studies

|  | **Item No** | **Recommendation** | **Fulfilled** | **cf.** |
| --- | --- | --- | --- | --- |
| **Title and abstract** | 1 | (*a*) Indicate the study’s design with a commonly used term in the title or the abstract | × | P1-P2 |
|  |  | (*b*) Provide in the abstract an informative and balanced summary of what was done and what was found | × | P2 |
| Introduction | | |  |  |
| Background/rationale | 2 | Explain the scientific background and rationale for the investigation being reported | × | P3-P4  L46-L88 |
| Objectives | 3 | State specific objectives, including any prespecified hypotheses | × | P4  L89-L95 |
| Methods | | |  |  |
| Study design | 4 | Present key elements of study design early in the paper | × | P5  L97-L105 |
| Setting | 5 | Describe the setting, locations, and relevant dates, including periods of recruitment, exposure, follow-up, and data collection | × | P5  L97-L105  P5-P6  L106-L124 |
| Participants | 6 | (*a*) Give the eligibility criteria, and the sources and methods of selection of participants. Describe methods of follow-up | × | P5-P6  L106-L124 |
|  |  | (*b*) For matched studies, give matching criteria and number of exposed and unexposed | × | P10  L221-L224  Table S7 |
| Variables | 7 | Clearly define all outcomes, exposures, predictors, potential confounders, and effect modifiers. Give diagnostic criteria, if applicable | × | P6-P7  L125-L164  P5  L112-L115 |
| Data sources/ measurement | 8* | For each variable of interest, give sources of data and details of methods of assessment (measurement). Describe comparability of assessment methods if there is more than one group | × | P7  L146-L164 |
| Bias | 9 | Describe any efforts to address potential sources of bias | × | P6  L121-L124 |
| Study size | 10 | Explain how the study size was arrived at | × | P7-P8  L166-L172 |
| Quantitative variables | 11 | Explain how quantitative variables were handled in the analyses. If applicable, describe which groupings were chosen and why | × | Tables 1, S4, S5, S6, S7 |
| Statistical methods | 12 | (*a*) Describe all statistical methods, including those used to control for confounding | × | P8-P10  L173-L227 |
|  |  | (*b*) Describe any methods used to examine subgroups and interactions  *The method for testing interactions is described. Subgroup analyses are conducted for chikungunya virus-infected subjects* | × | P9  L196-L214  Tables 2, 3  S4, S5, S6 |
|  |  | (*c*) Explain how missing data were addressed | × | P10  L226-L227 |
|  |  | (*d*) If applicable, explain how loss to follow-up was addressed | × | P6  L124 |
|  |  | (*e*) Describe any sensitivity analyses | × | P9-P10  L215-224 |
| Results | | |  |  |
| Participants | 13* | (a) Report numbers of individuals at each stage of study—eg numbers potentially eligible, examined for eligibility, confirmed eligible, included in the study, completing follow-up, and analysed | × | Flow chart Fig.1, Fig.S1 |
|  |  | (b) Give reasons for non-participation at each stage | × | Fig.1, Fig.S1 |
|  |  | (c) Consider use of a flow diagram | × | Fig.1, Fig.S1 |
| Descriptive data | 14* | (a) Give characteristics of study participants (eg demographic, clinical, social) and information on exposures and potential confounders  *Data are population-based estimates and the study was not conceived for assessing detailed demographic, clinical, social characteristics, or detailed information on exposures and confounders but merely to capture critical information on common outcomes* | × | Table 1 |
|  |  | (b) Indicate number of participants with missing data for each variable of interest  *All participants have information on disease outcomes* | × | Fig.1 |
|  |  | (c) Summarise follow-up time (eg, average and total amount)  *Given this is a reanalysis of the TELECHIK study, follow-up time is also specified in the method section* | × | P5  L107-L09  Table 1 |
| Outcome data | 15* | Report numbers of outcome events or summary measures over time  *Numbers are population-based percentages and prevalence ratios* | × | Tables 2,3 S4,S5,S6,S8 |
| Main results | 16 | (*a*) Give unadjusted estimates and, if applicable, confounder-adjusted estimates and their precision (eg, 95% confidence interval). Make clear which confounders were adjusted for and why they were included | × | Tables 2,3 S4,S5,S6,S8 |
|  |  | (*b*) Report category boundaries when continuous variables were categorized | N.A | - |
|  |  | (*c*) If relevant, consider translating estimates of relative risk into absolute risk for a meaningful time period | N.A | - |
| Other analyses | 17 | Report other analyses done—eg analyses of subgroups and interactions, and sensitivity analyses | N.A | - |
| Discussion | | |  |  |
| Key results | 18 | Summarise key results with reference to study objectives | × | P14  L299-314 |
| Limitations | 19 | Discuss limitations of the study, taking into account sources of potential bias or imprecision. Discuss both direction and magnitude of any potential bias | × | P17  L372-L389 |
| Interpretation | 20 | Give a cautious overall interpretation of results considering objectives, limitations, multiplicity of analyses, results from similar studies, and other relevant evidence | × | P7  L315-L371 |
| Generalisability | 21 | Discuss the generalisability (external validity) of the study results | × | P17  L389-L396 |
| Other information | | |  |  |
| Funding | 22 | Give the source of funding and the role of the funders for the present study and, if applicable, for the original study on which the present article is based | × | P10  L229-L235 |
| *Give information separately for exposed and unexposed groups. | | | | |
| **Note:** An Explanation and Elaboration article discusses each checklist item and gives methodological background and published examples of transparent reporting. The STROBE checklist is best used in conjunction with this article (freely available on the Web sites of PLoS Medicine at http://www.plosmedicine.org/, Annals of Internal Medicine at http://www.annals.org/, and Epidemiology at http://www.epidem.com/). Information on the STROBE Initiative is available at http://www.strobe-statement.org. | | | | |
